# A 13-YEAR TREND OF THE ABUSE OF OLDER PEOPLE IN SOUTH KOREA

**DOI:** 10.1093/geroni/igad104.2126

**Published:** 2023-12-21

**Authors:** Jenny Kwon

**Affiliations:** Miami University, Oxford, Ohio, United States

## Abstract

Compared to the growing number of the older population in South Korea, there is a scarcity of literature investigating issues about the abuse of older people, as well as effective prevention programs. To fill this gap, this study aimed to identify annual changes in patterns of the abuse of older people including the number of reported abuse cases; the type of abuse; and the characteristics of the subject. Data were drawn from the Korean Statistical Information Service (KOSIS), the public national statistical database. The present study used 13-year data during the period of 2008-2020. Descriptive analyses were conducted via SPSS. The number of abuse cases has increased every year from 2,369 in 2008 to 6,259 in 2020 and 10% of the reported cases in 2020 were repeated cases. The most common type of abuse reported was psychological abuse followed by physical abuse and neglect over 13 years. Physical abuse increased substantially from 22.4% to 40.0%. The majority of subjects are consistently women, accounting for 68.4% in 2008 and 75.3% in 2020. One in fourth of the subjects are older people living with dementia in 2020. Considering the serious impact of abuse including depression and suicide, abuse of older people is no longer a single individual or family problem. To protect the older population and improve their rights in society, collaborative efforts are critical. Micro-level movements such as increasing social awareness and national-level initiatives such as implementing policies and developing relevant legislation should be necessary.

